# Access to CPAP treatment in patients with moderate to severe sleep apnea in a Latin American City

**DOI:** 10.5935/1984-0063.20180032

**Published:** 2018

**Authors:** Juan Facundo Nogueira, Guido Simonelli, Vanina Giovini, María Florencia Angellotti, Eduardo Borsini, Glenda Ernst, Carlos Nigro

**Affiliations:** 1 Instituto Argentino de Investigación Neurológica, Sleep Laboratory - Buenos Aires - Ciudad Autonoma de Buenos Aires - Argentina.; 2 Hospital de Clínicas, University of Buenos Aires, Sleep Laboratory - Buenos Aires - Ciudad Autonoma de Buenos Aires - Argentina.; 3 Grupo Argentino de Investigación en Apneas del Sueño, Cooperative Group of Research - Buenos Aires - Ciudad Autonoma de Buenos Aires - Argentina.; 4 Walter Reed Army Institute of Research, Behavioral Biology Branch - Silver Spring - Maryland - United States.

**Keywords:** Sleep Apnea, Obstructive, Continuous Positive Airway Pressure, Compliance, Education

## Abstract

**Introduction:**

The most effective treatment for moderate to severe obstructive sleep apnea (OSA) is continuous positive airway pressure (CPAP) but adherence may be a limiting factor. Most compliance studies often only include patients under CPAP treatment, neglecting the importance of access to treatment. The aim of this study was to evaluate CPAP access and compliance in OSA patients, after CPAP indication and titration.

**Methods:**

We included moderate to severe OSA patients, diagnosed by in-lab polysomnography (PSG), with CPAP indication and effective pressure titration. Between 12 to 18 months after treatment was indicated a telephone questionnaire was administered including questions about access to CPAP, reasons of no access, reported adherence and symptoms improvement.

**Results:**

A total of 213 patients responded to the survey (171 males, mean age 53.4±13.5 and BMI 34.02±8.8 kg/m^2^). Almost a third of the patients (28.2%) did not initiate CPAP treatment. Out of 213, 153 patients (71.8%) started treatment with CPAP and 120 (56.3%) reported still being under treatment a year after indication, additionally, 85.8% reported that they were using it =4hs/night. Those who accessed to CPAP were on average, older age, had full coverage of treatment by their medical insurance, required lower effective pressure and experienced more severe sleepiness compared to those individuals who did not accessed to CPAP.

**Discussion:**

A significant proportion of OSA patients with CPAP indication did not initiate and/or eventually abandoned CPAP. Approximately only 50% of the patients were still under treatment, with acceptable self-reported adherence rate and clinical response, one year after the initial treatment indication. Additional measures are necessary to increase access to CPAP and improve long-term compliance.

## INTRODUCTION

Obstructive Sleep Apnea (OSA) is defined by the recurrent episodes of collapse of pharynx during sleep, associated to a fall in arterial oxygen saturation (SaO_2_), an increase of arterial carbon dioxide (pCO_2_) and arousals with sleep fragmentation^1^^-^^3^. As a consequence of OSA, patients report excessive daytime sleepiness and poor quality of life^1^^-^^3^. On the other hand, OSA is a risk factor for developing arterial hypertension (HTN)^1^^-^^5^ and cardiac arrhythmias^1^^-^^3^^,^^6^^,^^7^. Further, OSA can worse the evolution of patients with heart failure and coronary disease^8^^,^^9^. OSA is also associated with increased incidence of stroke^10^ and risk of motor vehicle accidents^11^. There is a growing evidence linking OSA with the development of metabolic syndrome and type 2 diabetes^12^^,^^13^.

The initial reported prevalence for OSA was estimated between 3.1-7.5% for men and 1.2-4.5% in pre-menopausal women, with similar estimates to those in men in post-menopausal women^1^^,^^3^^,^^14^. Recent estimates, however, have revealed higher prevalence of moderate to severe OSA, of approximately 9% for women and 24% for men^15^^,^^16^. Perhaps illustrative of the burden of OSA, the Hypnolaus study, reported prevalence of 49%, suggesting that OSA is a major public health threat, and with a much higher prevalence than originally reported^17^.

Continuous positive airway pressure (CPAP) treatment reverts upper airway (UA) collapse, suppressing snoring, obstructive events, oxygen desaturations and arousals, improving sleep quality and drowsiness. There is some evidence that CPAP use also reduces the risk of motor vehicle accidents and improves the management of hypertension, arrhythmias and type 2 diabetes^1^^,^^3^^,^^18^^-^^21^. As with many other chronic conditions, treatment compliance rates are a big concern. In OSA patients, without undergoing any other specific intervention, adherence to CPAP is estimated to 50%^22^^,^^23^. Some strategies, such as those geared towards education of the patient, training and dedicated follow-up, can significantly improve adherence and compliance to CPAP considerably^24^^-^^26^.

The majority of the studies evaluating compliance with CPAP only recruited patients who have started treatment, which could potentially overestimate compliance rates by excluding those who did not access CPAP treatment. Access to CPAP therapy has not yet been sufficiently addressed, especially in low and middle-income countries, such as in Latin America.

In a previous study, analyzing a similar population, 48% of moderate-severe OSA patients did not start treatment with CPAP and only 40% continued using it after 6 to 12 months of diagnosis^27^. That study showed that the lack of a precise indication by their family doctor and the patient’s unawareness about the importance of their illness and treatment were the most relevant factors associated with a lower adherence^27^.

The main objective of this study was, therefore, to determine the rate of moderate to severe OSA patients who access treatment and continue using CPAP after 12 to 18 months of the indication and effective pressure titration. The secondary objective was to determine the potential access to treatment barriers in patients that did not initiate the therapy, as well as the factors associated with the abandonment of treatment in those who choose to discontinue CPAP use.

## MATERIALS AND METHODS

This was an observational and prospective study. Patients were interviewed 12 to 18 months after the indication of CPAP treatment and were asked about access to treatment and compliance.

This investigation was conducted in accordance with the Declaration of Helsinki and approved in advance by the independent ethics committee of the Instituto Argentino de Investigación Neurológica (IADIN). Patients gave written informed consent prior to participate in the study.

### Study Sample

Our study sample was drawn from the pool of patients that were referred to the sleep lab of IADIN for a polysomnography (PSG) under the suspicion of OSA, consecutively between the months of January of 2012 and January of 2013. These patients were adults and all came from either the City of Buenos Aires or the Buenos Aires metropolitan area. Only patients with an apnea hypopnea index (AHI) ≥ 15 events/hour were recruited for this study. Age, gender, type of health insurance coverage (through work or private insurance) and body mass index (BMI) were recorded during their first visit to the sleep clinic. The degree of daytime sleepiness (EDS) was also assessed during this visit using the sleepiness scale (absent, mild, moderate, severe) proposed by the American Academy of Sleep Medicine (AASM)^28^.

### OSA Diagnosis

All the patients underwent a full-night PSG, using a digital system (Vertex, Pentatek, Argentina) at the sleep laboratory, during the subject’s habitual sleep schedule. The following parameters were monitored simultaneously and continuously: three channels of Electroencephalogram (EEG: F4, C4, O2); two channels of electrooculogram, three channels for the surface electromyogram (submentonian region, anterior tibialis muscle in both legs); one channel for an electrocardiogram; airflow detection via two channels through a thermocouple (one channel) and nasal pressure (one channel); respiratory effort of the thorax (one channel) and of the abdomen (one channel) using piezoelectric sensors; snoring (one channel) and body position (one channel); oxy-hemoglobin saturation (SpO2); and pulse rate. Two expert physicians visually scored all PSGs using standardized criteria^29^.

Obstructive sleep apnea was defined if there was a drop in the peak flow signal excursion by ≥ 90% of pre-event baseline (oronasal thermal sensor or an alternative apnea sensor) with persistent respiratory effort, for ≥ 10 seconds. Hypopnea was defined as a ≥ 30% drop in flow signal (nasal pressure) for ≥ 10 seconds, associated with ≥ 3% desaturation or with an arousal^29^. The severity of OSA was calculated on the basis of the patient’s AHI (number of apneas and hypopneas per hour), and a AHI greater than 15 was classified as moderate-severe^1^^,^^2^. The decision to indicate CPAP treatment followed the criteria proposed by the regional medical guidelines in accordance with AASM guidelines^1^^,^^21^.

### CPAP training and titration

After moderate to severe OSA was diagnosed, a staff physician interviewed the patient, explaining the illness implications and the rationale and benefits of CPAP treatment. After this medical visit, patients were scheduled for CPAP testing and training and mask fitting at the sleep clinic. This intervention consisted of a 40-60 minutes session with an expert technician. Possible side effects of CPAP were explained again in detail. Each patient took home an auto-CPAP device (Autoset S9, ResMed) and the mask chosen (among different types and models) to use it for CPAP titration during six nights.

They also had to complete a sleep diary. Patients were encouraged to contact the expert technician if there were problems or doubts related to CPAP by cellular phone at any time. Objective data from every device was collected after this six-days trial, mean and 95^th^ percentile CPAP pressure, effective pressure curve, nightly usage, leak and residual AHI were evaluated in order to determine the effective pressure for CPAP treatment^1^^,^^3^^,^^30^^,^^31^. Titrations were considered *optimal* if the patients used auto-CPAP on average at least 4hs per night, without significant leak (defined as >24 LPM during ≥30% of usage time) and a residual AHI (measured by the device) ≤5 ev/h. Titrations were considered *acceptable* if the residual AHI was ≤10 ev/h or ≤75% of basal AHI in severe OSA patients, with the same criteria for leak and time of use. Titrations that did not meet these criteria were repeated^30^^,^^31^.

### CPAP indication

Finally, the patients received a written indication for CPAP that included their preferred mask and effective pressure.

### Follow up

Our Sleep Laboratory is a reference center for sleep studies of different private and workers health insurance companies, many patients are referred for diagnostic procedures, but we do not follow all of them after the indication of treatment. Therefore, with the intention of evaluating the entire population of patients diagnosed in that period of time, between 12 and 18 months after CPAP indication subjects were interviewed via telephone by trained staff. During this interview patients were asked about access to CPAP therapy (Have they accessed CPAP treatment?), compliance (Were they using it? How many hours per night? How many nights per week?), reasons why they did not access or were not compliant with the treatment if applicable. Adherence was not objectively measured. Patients under CPAP treatment were also asked whether they had experienced any improvement (none, mild, moderate or great improvement) with their snoring, sleepiness, resting at night and cognitive performance.

### Data Analysis

Sample characteristics are shown as mean (SD) or number (%). Patients were divided into three different categories for analytical purposes: patients that did not access CPAP treatment, patients that initiated and interrupted CPAP therapy and those who accessed CPAP and continued to be under treatment. For the last group, they were considered compliant with the treatment if they used CPAP ≥ 4 hours/night, at least 5 nights a week according to current guidelines^1^^,^^3^. Differences between the group that accessed CPAP treatment and the group who did not accessed CPAP were assessed with X² for categorical variables (sex, EDS and medical coverage) and T test or Mann-Withney for continuous variables (age, AHI, BMI). A statistically significant difference was set at *p*<0.05. Medcalc and Graph Pad Prism-5 software were used for the analysis.

## RESULTS

**Figure 1:**
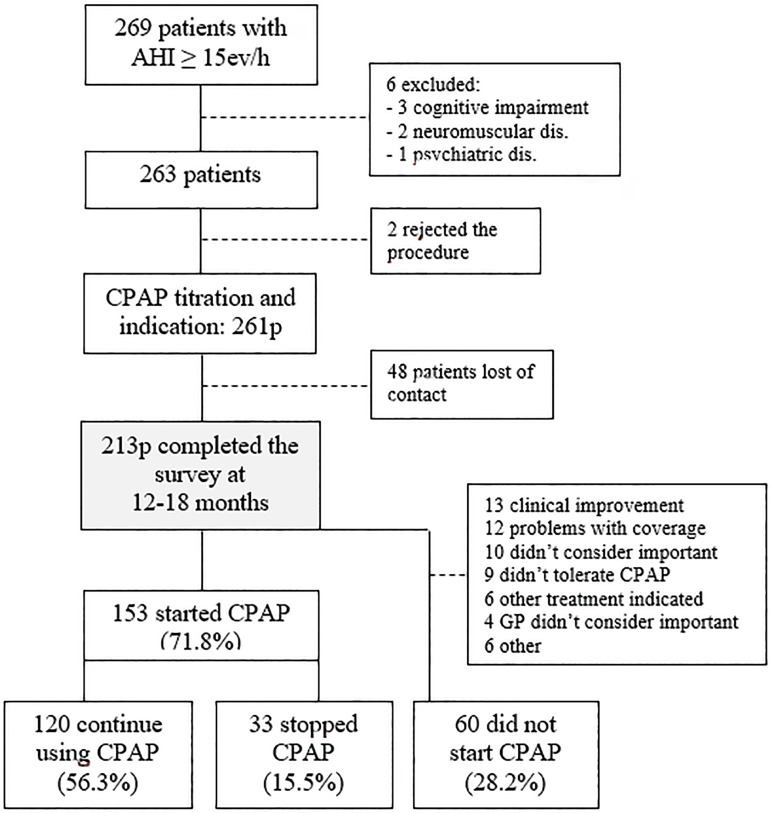
Flowchart of patients. AHI: apnea hypopnea index; CPAP: continuous positive airway pressure

A total of 269 patients were recruited for this study. Of these, 6 were excluded due to severe illness, 2 rejected CPAP titration following OSA diagnosis and 48 could not be reached via telephone. In terms of age, gender, BMI and AHI, there were no statistical differences between those who completed the interview and those who did not (Table 1). Figure 1 shows the flow diagram of excluded and included patients. We do not have the follow-up data on these 48 patients. We cannot infer whether or not they were adherent to CPAP treatment. In any case, the response rate was 79.2%, sufficiently high considering similar studies^27^^,^^32^^,^^33^.

**Table 1:** Differences among patients who completed survey vs. those who did not.

N: 269	Recruited	Not included	*p* value
N	213 (79.2%)	56 (20.8%)	
Males	171 (80.3%)	44 (78.6%)	Ns
Age (y/o)	53.4±13.5	53.2±13.2	Ns
BMI (Kg/m^2^)	34.02±8.8	33.9±8.6	Ns
AHI (ev/h)	49.01±18.7	47.9±18.4	Ns

AHI: apnea hypopnea index; BMI: body mass index.

Table 2 shows the sample characteristics. The mean age of the interviewed patients was 53.4±13.5; 171 were men (80.3%); the BMI was 34.02±8.8 and 148 (69.5%) were obese. The mean AHI was 49.1±18.7 ev/h and the mean titrated CPAP pressure was 8.93±2.05cmH_2_O. Nasal masks were preferred by 165 (77.4%) patients. Our results show that 153 patients (71.8%) started CPAP treatment and 60 patients (28.2%) did not. Of those who started CPAP treatment, 120 were still using it at the time of the interview, with an average of 6.38±1.85 hs/night, during 6.37±1.38 nights a week, additionally, 108 reported a use of at least 4 hours per night, ≥ 5 nights a week (Figure 1).

**Table 2:** Characteristics of the population.

N	213
Males	171 (80.3%)
Age	53.4±13.5 years
BMI	34.02±8.8 kg/m^2^
Obese (BMI >30)	148 (69.5%)
AHI	49.01±18.7 ev/h
CPAP pressure	8.93±2.05 cmH_2_O
Workers Health insurance	152 (71.4%)
Private Health insurance	61 (28.6%)

AHI: apnea hypopnea index; BMI: body mass index; CPAP: continuous positive airway pressure

Of the total number of participants who reported having started CPAP treatment, approximately one fifth (33 patients) stopped the treatment. The causes for these patients to stop using CPAP were diverse: 33.3% expressed “feeling better”; 27.3% patients did not tolerate using CPAP; 9.1% reported the presence of adverse events as the main cause of stopping the treatment and 30.3% expressed “other” as the reason to interrupt therapy (Table 3). The patients who never started CPAP treatment were 60, over a quarter of the total sample. The main reasons for patients not to start CPAP treatment are shown in Table 3.

**Table 3:** Causes of non-CPAP use.

A) Causes of stopping treatment. N: 33p.	
Clinical improvement	11 (33.3%)
CPAP intolerance	9 (27.3%)
Adverse events	3 (9.1%)
Other	10 (30.3%)
B) Causes of no starting treatment. N: 60p.	
Clinical improvement	13 (21.6%)
Problems with coverage	12 (20%)
Patient did not consider it necessary	10 (16.6%)
CPAP intolerance	9 (15%)
Other indication	6 (10%)
Family doctor did not consider it necessary	4 (6.6%)
Other	6 (10%)

CPAP: continuous positive airway pressure.

Overall patients who did not start the treatment reported the following main reasons: feeling better (21.6%), not having coverage for the treatment (20%), feeling that OSA was not a disorder that needed treatment (16.6%), not tolerating the CPAP during the titration (15%) and family doctor dismissing CPAP indication or indicating an alternative treatment (16.6%). Among those patients who were not under CPAP treatment, in only 9 an alternative treatment was implemented (7 upper-airway surgery, 2 mandibular advancement device) and bariatric surgery was performed in 5 subjects.

**Figure 2:**
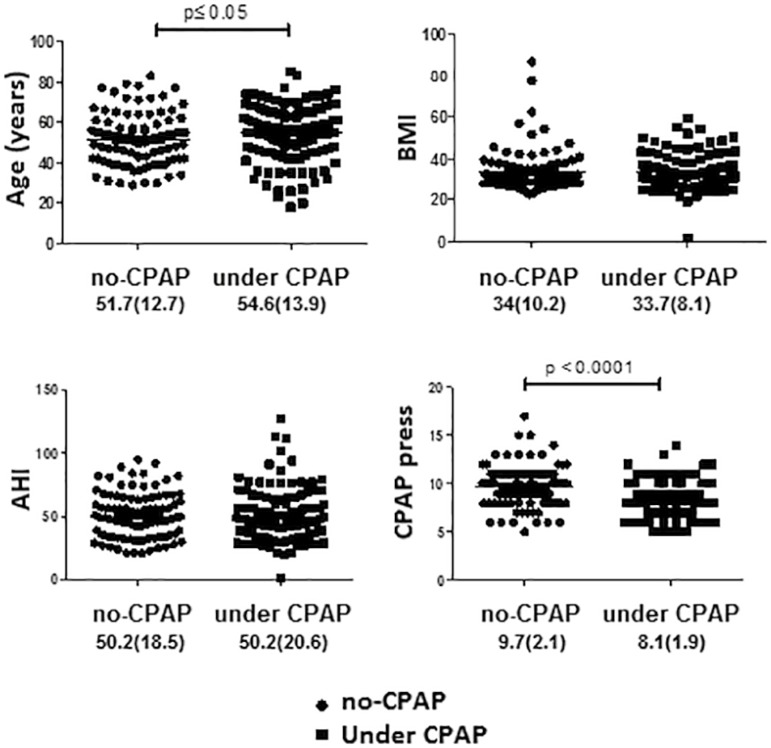
Comparison of patients under CPAP vs. no-CPAP. CPAP: continuous positive airway pressure.

**Figure 3:**
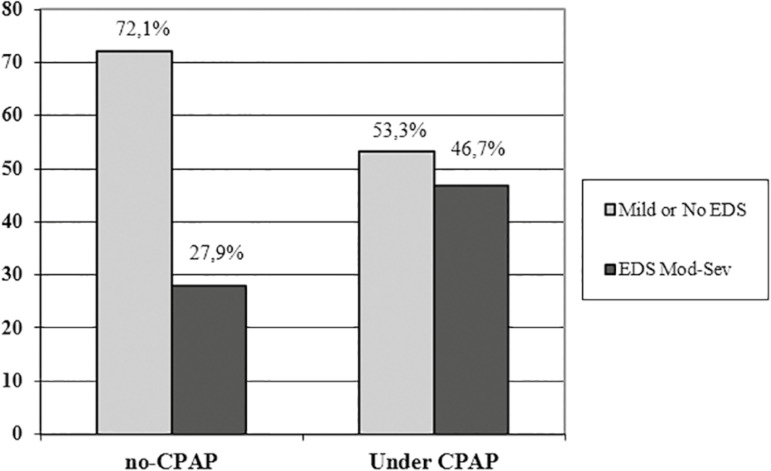
Percentage of patients with EDS moderate-severe vs. EDS mild or no-EDS, according to the patients were under CPAP or not.CPAP: continuous positive airway pressure; EDS: excessive daytime sleepiness

Table 4 shows the comparison between the patients who reported using CPAP and the patients who did not. Patients that used CPAP were significantly older (*p*≤0.05), required less positive pressure CPAP (*p*≤0.001) (Figure 2) and had higher basal EDS compared to those who did not use CPAP (Figure 3). No differences were found in terms of gender and AHI between these two groups.

**Table 4.** Comparison of patients under CPAP vs. no-CPAP.

	Under CPAP	no-CPAP	*p* value
N	120	93	
Age (years)	54.6±13.9	51.7±12.7	≤ 0.05
BMI (k/m^2^)	33.7±8.1	34±10.2	ns
Males	78.3%	82.8%	ns
AHI (ev/h)	50.2±20.6	50.2±18.5	ns
CPAP pres (cmH_2_O)	8.1±1.9	9.7±2.1	≤ 0.0001
Nasal mask	94 (78.3%)	71 (76.3%)	ns
Mod-Sev EDS	56 (46.6%)	26 (27.9%)	≤ 0.02

AHI: apnea hypopnea index; BMI: body mass index; CPAP: continuous positive airway pressure; CPAP pres: effective CPAP pressure defined by titration.

**Figure 4:**
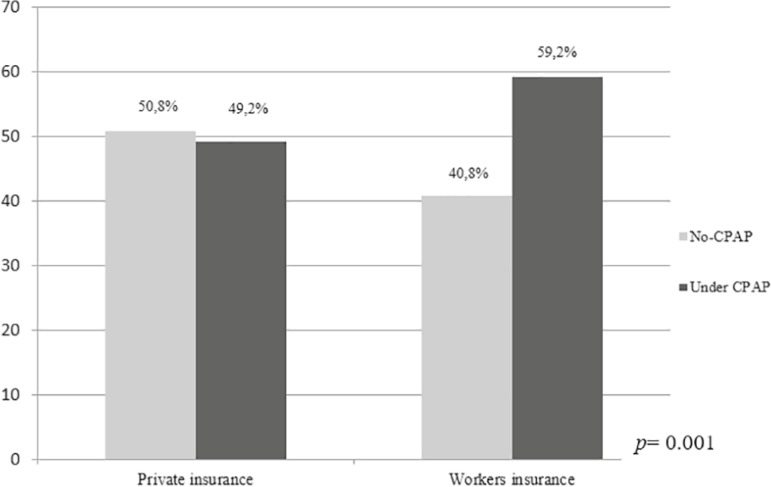
Access to CPAP treatment among patients from private versus workers health insurance. CPAP: continuous positive airway pressure.

All patients had some type of medical coverage; 61 (28.6%) had private health insurance and 152 (71.4%) had insurance through their employment. Private insurance partially covered CPAP treatment (50% of the cost) whereas, employers insurance covered the total cost of CPAP treatment. When dividing the sample into those who had partial coverage and total coverage, we found that 59.2% of patients who had full coverage for CPAP treatment initiated treatment versus 49.2% that initiated treatment with partial coverage (*p*=0.001) (Figure 4).

Finally, when patients under CPAP were asked about the evolution of symptoms after treatment, moderate or great improvement were reported in 79.8% of patients for snoring, in 86.5% for sleepiness, in 92.5% for resting at night and in 87.4% for cognitive performance.

## DISCUSSION

Our results show that less than sixty percent of patients with moderate to severe OSA were under CPAP treatment after one year of indication and almost a third of the patients with moderate to severe OSA had not accessed CPAP treatment at all. These findings are consistent with two previous studies, one from Canada and one from China, which showed that approximately 30% of OSA patients do not access CPAP treatment^32^^,^^34^. A third similar study from Mexico, revealed that 34.8% of patients did not access positive pressure therapy after indication^35^. Other authors reported values between 72-79% for continuing use at 1 year, but the analyses were not performed on an “intention to treat” basis^21^^,^^36^^,^^37^. Among patients under CPAP treatment, 85.5% reported adequate compliance. It is worth noting that this information was self-reported and may not accurately reflect reality, as patients tend to overestimate the numbers of hours of CPAP use^22^.

Approximately 15% of the patients in this sample initiated and subsequently abandoned the treatment within one year after the CPAP indication. These results are not surprising and are consistent with previous work^27^^,^^32^^,^^34^^,^^38^. CPAP intolerance and development of adverse effects were the main reasons for discontinuation of treatment. Additionally, a third of our sample reported that they abandoned the treatment because they felt better. From our perspective, this behavior is especially concerning, since it could indicate some degree of denial or underestimation of the disorder or even, if they had become asymptomatic, OSA with or without symptoms is also detrimental to health^39^.

Our study differed significantly from previous studies evaluating CPAP compliance in two important ways. First, we assessed compliance differently by calculating not only the rate of use among patients who started CPAP therapy, but also by determining the proportion of patients originally indicated CPAP who, for different reasons, did not to initiate treatment. Second, we evaluated long-term compliance, a minimum of one year after initial diagnosis, not just a few months after indication. We consider that these two aspects constitute key elements to determine compliance with CPAP treatment in a real life scenario.

In our study, patients that acceded and adhered to CPAP treatment were older than the ones who did not. These findings are consistent with previous studies^33^^,^^40^^-^^44^; while other investigators did not find association between age and adherence to CPAP^22^^,^^32^^,^^35^^,^^45^^-^^48^ and some even reported an opposite correlation^36^^,^^38^. Although the design of this study did not allow exploring the causes of lower adherence in younger patients, there are some potential explanations for these findings. For instance, the use of the device for sleeping may constitute a barrier in younger patients with a more active social life; in other words, the use of a CPAP device may be less disturbing for social life of the elderly^33^^,^^40^. Additionally, retirement implies more hours available for sleeping and less stressful life, compared to active working age adults; in large cities, such as Buenos Aires, the greater use of CPAP could be explained by more time to sleep^24^. At last, it can be speculated that our finding could be related to a greater number and severity of cardiovascular comorbidities at older ages, which would result in a greater incentive to comply with treatment^33^.

Patients who were under CPAP had higher basal EDS compared to those not on treatment. This finding was previously described^32^^,^^33^^,^^38^^,^^46^^,^^48^^-^^50^. The most severely (higher degree of EDS) ill OSA patients are the ones who have additional motivation to obtain a unit, being more symptomatic may stimulate patients to adhere to therapy as it affects their quality of life, symptoms may increase awareness of severity and improvement in symptoms with CPAP may reinforce the compliance to treatment^41^^,^^50^^-^^52^. Conversely, other authors have failed to find association between CPAP adherence and EDS severity^24^^,^^27^^,^^35^^,^^40^^,^^44^.

We did not find differences in OSA severity (AHI) between individuals who had access and adhered to CPAP, in contrast to what other studies have reported^22^^-^^24^^,^^32^^,^^40^^,^^41^^,^^43^^,^^44^. Patients who were not under CPAP treatment after 12-18 months of treatment indication required higher CPAP pressure at titration study. An explanation for this might be that higher CPAP pressures may be more difficult to tolerate. It would be reasonable to expect that increased pressure increases mouth leakage and nasal symptoms, two factors associated with decreased compliance. In the Pelletier-Fleury’s study, the patients treated with a pressure of 12 cmH2O or greater were 2.3 times less compliant than the patients treated with a lower pressure^48^.

Some studies comparing fixed versus intelligent CPAP have shown greater adherence to treatment associated to a lower mean CPAP pressures with the use of a self-adjusting device^53^^,^^54^. Nevertheless, although some authors reported the same finding^46^^,^^48^, others found an inverse relationship^38^^,^^47^ or found no association between effective CPAP pressure and adherence^23^^,^^24^^,^^32^^-^^35^^,^^44^^,^^49^^,^^51^. These observations should be confirmed in a larger cohort of patients. Undoubtedly, there are unknown factors that may significantly affect both access and adherence to CPAP, and it is probably that our ability to understand and control compliance is limited in the context of chronic treatments^35^.

Private insurance could be considered a proxy of a higher socioeconomic status, but, in this population, private insurance partially covered CPAP treatment (50% of the cost) meanwhile, employers insurance covered the total cost of CPAP treatment. Access to CPAP was lower in patients with partial medical coverage compared to those with full coverage of therapy, a result that may indirectly suggests that the cost of equipment may be a barrier of access. Treatment coverage seems to be a particularly important factor associated with access to CPAP. For example, one Mexican study showed that treatment coverage was the main limiting factor for access to CPAP^35^.

In contrast, studies performed in the context of health care systems, such as the ones in many European countries show that access to CPAP treatment can be as high as over 95%^38^. Even in the context of full CPAP treatment coverage, socioeconomic status remains an important predictor of treatment, and low socioeconomic status is associated with greater rejection to treatment compared to individuals with higher socioeconomic status^42^. It is possible, as it has been highlighted before, that the wide variability of published results may be a reflection and/or consequence of different criteria for patient selection, sample size or statistical analysis of the data^48^.

Approximately 80% of patients under CPAP treatment reported substantial improvements in snoring, sleepiness, resting at night and cognitive performance. On the other hand (and unfortunately), we did not inquire about symptoms improvement to those who were not being treated.

Our study has some limitations. First, we had no treatment information about the patients who did not participate in the survey, although the response rate was relatively high. A second limitation is that this study was conducted in a private sleep center, which provides assistance only to patients with some type of medical coverage, so we did not recruit patients without health coverage. In Argentina 30% of population does not have medical coverage, and according to previous reports, access to therapy could be even worse than our findings reflect^55^.

As an additional limitation, CPAP adherence was not evaluated using objective measures and the reasons for poor adherence relied on patients’ responses to our questions. Patients tend to overestimate compliance rate^22^, therefore, we could assume that the situation should be worse. However, our main objective was to evaluate access to CPAP in patients diagnosed with OSA, including all those who did not initiate or discontinue treatment and patients who, after being diagnosed and indicated treatment in our sleep center, were followed by another physician. For this reason, we consider that the design and source of information implemented were the most accessible and appropriate for our purposes. Nevertheless, the lack of stringent procedures to guarantee follow-up for these patients could have influenced the low rate of access to treatment. On the other hand, these results do show difficulties in accessing and adhering to CPAP treatment that should not be ignored.

Our study has a number of strengths as well. To our knowledge there are limited experiences in terms of access and adherence to CPAP treatment in South America^27^^,^^33^^,^^35^. This research shows data from a sleep center in Buenos Aires, a city with more than 15 million inhabitants and an under represented location in terms of sleep epidemiology. Our data might also reflect in some way, what many sleep clinics across Buenos Aires or even in other Latin American cities experience. At last, many previous studies have evaluated compliance over relatively brief periods of time (one to six months)^23^^,^^24^^,^^40^^-^^43^, whereas in our study, the time of follow up was greater than one year.

Additionally, as a remarkable aspect, our findings highlight the importance of not only addressing adherence among patient who had started CPAP treatment, but also evaluating access to therapy after indication. We determined compliance rates calculating not only how many hours patients use CPAP, but also by determining the proportion of patients that were originally indicated CPAP who were still using the device, including those who did not initiate treatment or abandoned it during the follow up period. This could be especially relevant in regions with limited treatment coverage.

## CONCLUSION

Compliance to CPAP is an important issue that may compromise treatment effectiveness. In this study one third of patients treated in a private sleep center in Buenos Aires did not access CPAP and an further 15% abandoned the treatment within the first year since CPAP indication; approximately one half of our patients reported being adherent to the treatment, coincidently with reports from other countries. Patients who do not access CPAP treatment represent a waste of scarce health resources that occurs when patients undergo expensive diagnostic procedures and then do not receive the needed treatment. Older age, greater sleepiness and full coverage of CPAP were factors identified in those who accessed treatment and reported being compliant compared to those were not.
